# Lipopolysaccharide‐induced sickness behavior is not altered in male *Fmr1*‐deficient mice

**DOI:** 10.1002/brb3.3142

**Published:** 2023-07-05

**Authors:** Danielle Santana‐Coelho, Samantha L. Hodges, Saul I. Quintero, Paige D. Womble, D. Greg Sullens, David A. Narvaiz, Rebecca Herrera, Melanie J. Sekeres, Joaquin N. Lugo

**Affiliations:** ^1^ Department of Psychology and Neuroscience Baylor University Waco Texas USA; ^2^ Institute of Biomedical Studies Baylor University Waco Texas USA; ^3^ Department of Biology Baylor University Waco Texas USA

**Keywords:** fragile X syndrome, lipopolysaccharide, sickness behavior, tail suspension, wheel running

## Abstract

**Objectives:**

Fragile X syndrome is the main monogenetic cause of intellectual disability and autism. Alterations in the immune system are commonly found in these developmental disorders. We and others have demonstrated that *Fmr1* mutant mice present an altered response to immune stimuli. However, whether this altered immune response can influence the *Fmr1* mutant behavioral outcomes in response to inflammation has not been fully investigated.

**Materials and methods:**

In the current study, we examine the behavioral sickness response of male wildtype and knockout  mice to the innate immune stimulus lipopolysaccharide (LPS) (0.1 mg/kg) to determine if *Fmr1* mutants have altered sickness behavior. We used an enzyme‐linked immunosorbent assay (ELISA) to measure changes in the cytokine interleukin‐6 (IL‐6) to determine that inflammation was induced in the mice. Sickness behavior was assessed in a wheel‐running paradigm, and a tail suspension test was used to assess the depressive‐like phenotype that follows sickness behavior in response to LPS.

**Results:**

The ELISA using blood serum confirmed a significant increase in IL‐6 in mice that were treated with LPS. Treated *Fmr1* mutants exhibited decreased distance traveled in the wheel running after LPS administration, similar to treated controls. Another cohort of animals treated with LPS were tested in the tail suspension test and exhibited no alterations in immobility time in response to LPS.

**Conclusion:**

Together, our data suggest that *Fmr1* mutant mice do not have altered sickness behavior in response to a low dose of LPS.

## Graphical abstract

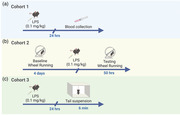

## 1 INTRODUCTION

Fragile X syndrome (FXS) is an X‐linked neurodevelopmental disorder that is caused by the functional loss of the fragile X messenger ribonucleoprotein 1 (*Fmr1*) gene, resulting in a reduction or absence of RNA‐binding protein fragile X messenger ribonucleoprotein (FMRP) (Hagerman, 2006; Hagerman & Hagerman, 2002). It is the most common monogenetic cause of autism spectrum disorder (ASD), with the disorders sharing several cognitive and behavioral phenotypes, including social communication deficits and stereotypical behavior (Harris et al., 2008). Evidence suggests that immune dysfunction could play a role in the etiology of both disorders (Ashwood et al., 2010; Eissa et al., 2020; Masi et al., 2017; Petrelli et al., 2016). The molecular and physiological basis of neuroimmune alterations in FXS and ASD has been studied extensively. However, whether dysregulated immune function could impact the behavioral phenotype observed in neurodevelopmental disorders is largely unknown.

A significant component of an immune response is the coordinated set of behavioral changes initiated by the insult, collectively known as sickness behavior (Dantzer et al., 2008). In response to peripheral infection, cells of the innate immune system release pro‐inflammatory cytokines that act on neural pathways in the brain and trigger a highly organized signaling cascade to fight infection (Layé et al., 1994). The key inflammatory cytokines known to be involved in the sickness response are interleukin 1 beta (IL‐1β), tumor necrosis factor alpha (TNFα), and interleukin‐6 (IL‐6) (Dantzer, 2009). Behavioral changes associated with the sickness response include lethargy, loss of appetite, sleepiness, social withdrawal, and depressed mood (Dantzer, 2001). While these adaptive behavioral changes are often viewed as unpleasant, they play a critical role in facilitating recovery from the infection.

Systemic administration of the endotoxin lipopolysaccharide (LPS), a component of the cell wall of Gram‐negative bacteria, is a classical way of mimicking a peripheral bacterial infection in rodent models (Bassi et al., 2012). It induces a peripheral immune response that is accompanied by similar neuroimmune and endocrine changes that mimic the human response to infection. The behavioral sickness response is similar in rodents, which includes fatigue, anhedonia, little to no interest in their physical and social environment, and rodents exhibiting a hunched posture often in the corner of their home cage (Hart, 1988). This response can also lead to a reduction in food and water intake by rodents. To investigate sickness behavior, several behavioral paradigms are used, including burrowing of food and sucrose preference tasks. Wheel running can also be utilized to examine changes in motor activity, another measure of the sickness response (Biesmans et al., 2013). LPS administration can also induce depressive‐like behavior at a later timepoint, characterized by increased immobility time in the tail suspension test and forced swimming test (Lasselin et al., 2020).

We and others have previously shown that *Fmr1* knockout (KO) mice in the Fvb background strain exhibit altered immune functions and responses to LPS (Hodges et al., 2020; Parrott et al., 2021). Immune regulation of behavior has been shown by several studies that demonstrate that inflammation can alter neurotransmission (Vezzani & Viviani, 2015). Neurotransmitter signaling can be regulated by inflammatory cytokines such as IL‐6. Exposure to IL‐6 downregulates serotonergic transporters (SERT) in cell culture, while IL‐6 KO mice have elevated levels of SERT and decreased depressive‐like behavior (Kong et al., 2015). IL‐6 can also regulate ion channel activity, such as Nav1.7 by increasing the number of spikes and decreasing the latency to the first action potential (Yan et al., 2012). Additionally, a large body of literature suggests that IL‐6 plays an important role in the pathophysiology of depression, where both in clinical and pre‐clinical studies, increased IL‐6 levels are associated with a depressive phenotype (Roohi et al., 2021).

Based on previous studies that have shown the role of IL‐6 in behavior (Kong et al., 2015; Sukoff Rizzo et al., 2012) and that have identified increased levels of IL‐6 in *Fmr1* KOs in response to LPS (Hodges et al., 2020; Parrott et al., 2021), we hypothesized that *Fmr1* KOs will present with more severe sickness behavior and depressive‐like phenotypes than wild‐type (WT) mice. Hence, in the present study, we examined the behavioral sickness response in a mouse model of FXS, utilizing *Fmr1* KO and WT mice on a C57BL/6J background strain. Following a period of baseline running, mice were administered either a single dose of the bacterial mimetic LPS (0.1 mg/kg) or of saline, followed by having access to running wheels for ∼2 days. Total voluntary wheel running activity, in addition to differences in the pattern of fatigue across the testing period, was examined between genotypes. To also examine potential depressive‐like behavior in *Fmr1* KO and WT mice, one cohort of mice was tested 24 h following LPS administration in the tail suspension task. This study will provide insight into whether a mouse model of FXS, a common neurodevelopmental disorder, displays an altered sickness behavioral response following an immune insult.

## 2 METHODS

### 2.1 Animals

Experimental subjects included male *Fmr1* KO and WT mice on a C57BL/6J background strain (Jackson Labs). We used only male mice based on our previous study that showed altered immune response in male *Fmr1* KO mice with the FvB/NJ background (Hodges et al., 2020). Mice used in the study were between 43 and 106 days old. The sample sizes for 24 h IL‐6 measurements were as follows: N = 15 for WT, N = 18 for *Fmr1* KO for the 24 h timepoint. The sample sizes for the wheel running mice were as follows: N = 24 for WT and *Fmr1* KO. The sample sizes for the 24 h tail suspension were as follows: N = 23 for WT, N = 27 for *Fmr1* KO. Breeding pairs consisted of male WT sire and *Fmr1* Het dams. Mice were bred and housed at Baylor University in standard laboratory conditions (22°C, 12 h light/dark cycle) and had ad libitum access to food and water throughout the experiment. All procedures were performed in compliance with the Baylor University Institutional Animal Care and Use Committee and the *National Institute of Health Guidelines for the Care and Use of Laboratory Animals*.

### 2.2 Lipopolysaccharide stimulation

The bacterial endotoxin LPS was administered to examine the effects of innate immune stimulation on sickness and depressive‐like behavior in *Fmr1* mutant and WT mice. LPS was prepared at a stock concentration of 1 mg/mL in 0.9% physiological saline and further diluted in saline prior to administration. All mice received either a single intraperitoneal injection of 0.1 mg/kg LPS from *Escherichia coli* serotype O127:B8 purified by gel‐filtration chromatography (Sigma) or an equivalent volume of filtered 0.9% physiological saline as a control. All injections were approximately 200 μL. The 0.1 mg/kg LPS dose was chosen based on preliminary wheel running experiments in C57BL/6J mice that demonstrated this as an ideal dose to examine the recovery from an LPS insult. Additionally, our previous study indicated that a higher dose of LPS (0.33 mg/kg) could induce a flooring effect in the sickness behavior test. The 0.1 mg/kg dose was used in the present study to potentially reveal any subtle behavioral differences between genotypes, while avoiding a potential floor effect with all mice exhibiting significant signs of sickness behavior.

**FIGURE 1:**
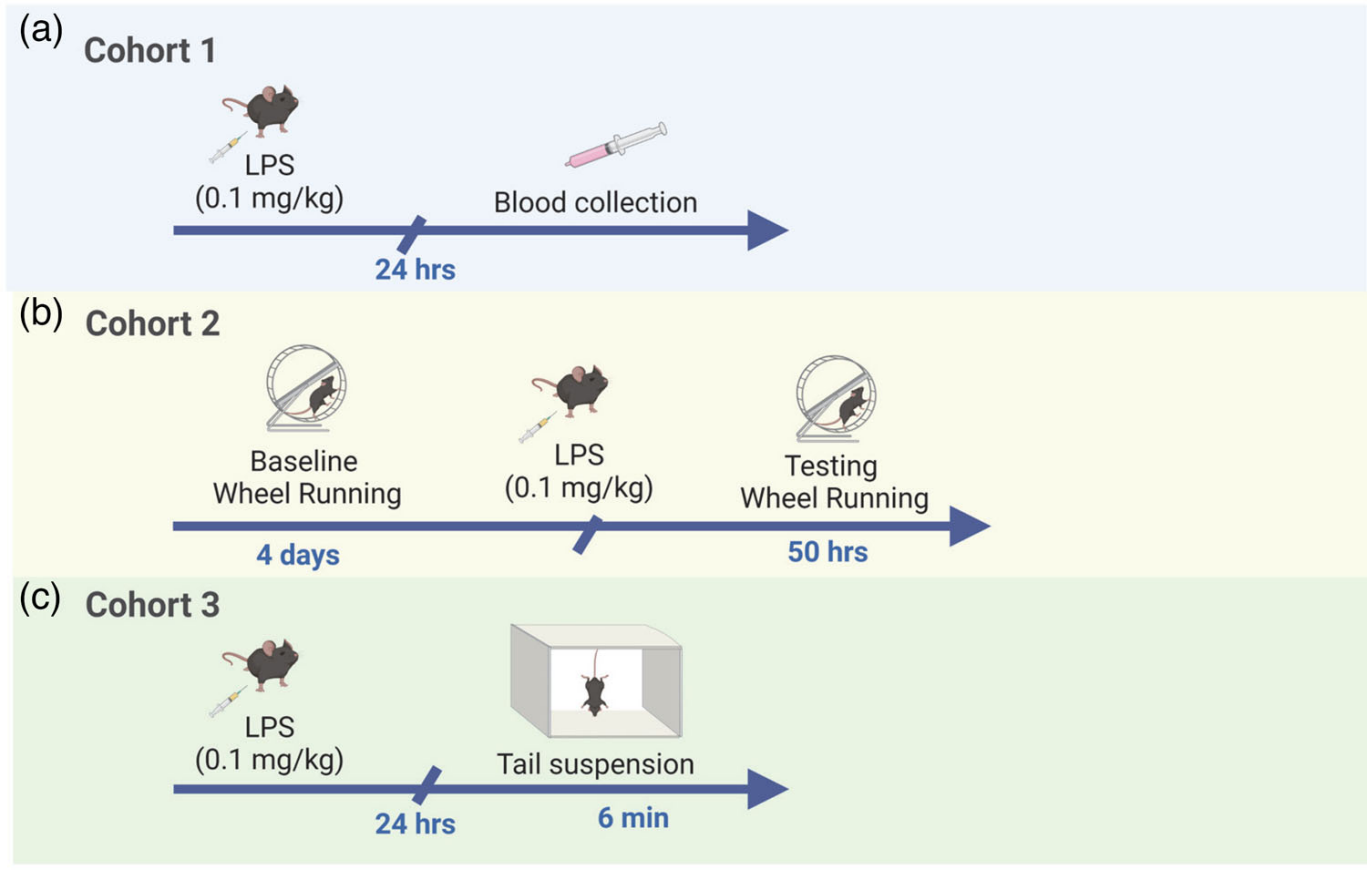
Experimental design. (a) In Cohort 1, mice were treated with lipopolysaccharide (LPS), and 24 h later blood was collected to measure the levels of interleukin‐6 (IL‐6). (b) In Cohort 2, baseline wheel running was recorded for 4 days. The animals were treated with LPS (0.1 mg/kg, intraperitoneal) and then were returned to the home cage. Wheel running was recorded for 50 h. (c) In Cohort 3, mice were treated with LPS, and 24 h after treatment, the tail suspension test was performed.

In the first cohort of mice, injections were administered at ∼12 p.m., and blood was collected 24 h later. In the second cohort of mice, injections were given at 12 p.m. following 4 days of baseline wheel running. Following injections, mice were placed back in their individual cages with a running wheel for approximately 50 h of testing. In the third cohort of mice, LPS injections were given at 12 p.m., and the tail suspension test was performed 24 h later (Figure 1).

### 2.3 Enzyme‐linked immunosorbent assay

The first cohort of animals was treated with LPS (0.1 mg/kg), and blood was collected 24 h after treatment to measure IL‐6 levels and confirm an immune response to the dose used in the study. Trunk blood was collected and an hour later centrifugated for 10 min at 1000 *g* at room temperature. Serum was isolated, frozen, and stored at −20C° until further analysis. IL‐6 was measured in serum samples using a commercially available mouse IL‐6 enzyme‐linked immunosorbent assay (ELISA) kit (ELISA MAX™ Deluxe Set, Biolegend) following the manufacturer's protocol. Data are expressed in pg/mL.

### 2.4 Voluntary wheel running

In the second cohort of animals, mice went through 4 days of baseline running prior to administration of LPS or saline, followed by a testing period of ∼2 days (50 h) in which mice were able to voluntarily run (Figure 1b). Throughout the duration of the experiment, mice were individually housed in a home cage with a free‐wheel running apparatus (ENV‐044 Mouse Low‐Profile Wireless Running Wheel, Med Associates Inc.; 15.5‐cm circumference; 25° from horizontal plan) placed inside the cage. Wheel rotations were continuously collected in 60 s intervals via wireless sensors that transmit wheel rotation signals to a monitoring hub (DIG‐804, USV interface hub). The number of revolutions was recorded with Wheel Manager Software Version 1.10 (SOF‐860). Revolutions were converted to total distance ran (centimeters) for each mouse during baseline and testing (1 revolution = 3.69 cm). Individual housing of subject mice was necessary in order to prevent competition for access to the running wheel and to monitor the running activity independently for each mouse.

### 2.5 Tail suspension

Tail suspension was conducted to examine depressive‐like behavior following administration of LPS in *Fmr1* mutant and WT mice (Figure 1c). Each mouse was suspended in a visually isolated area by fastening their tail to a padded close pin attached to an elevated shelf in a room controlled for background noise, temperature, and light levels. To prevent tail climbing behaviors, mouse tails were passed through a piece of cut straw (∼4 cm) prior to suspension. Total time spent moving during a 6‐min period was recorded by an experimenter blinded to the genotype of each subject. Immobility was calculated by subtracting the seconds the mouse was mobile from the total duration of the testing session (360 s). Measurements of immobility were defined as the absence of hind limb movement (Can et al., 2012; Cryan et al., 2005). Small movements confined to the front legs that did not involve the hind limbs, or swinging due to momentum gained during earlier mobility, were not counted as mobility.

### 2.6 Statistics

All statistical analyses were performed using GraphPad Prism 7 software (La Jolla) or SPSS 25.0 (IBM). The levels of IL‐6 in response to LPS were analyzed with a two‐way analysis of variance (ANOVA) to compare controls and *Fmr1* mutant IL‐6 levels. Changes in weight from the beginning of baseline wheel running, injection date, and tail suspension were analyzed with a two‐way repeated measures ANOVA, with a within‐subject factor of time and between‐subject factors of genotype and treatment. Tail suspension was analyzed with a two‐way ANOVA to examine differences in immobility across genotype and treatments. Average baseline wheel running was analyzed with a *t*‐test. Total wheel running following immune stimulation during the testing period was analyzed with a two‐way ANOVA. A two‐way repeated measures ANOVA with a within subject factor of time and between subject factors of genotype and treatment was also conducted to examine changes in running patterns over time after immune stimulation, followed by *t*‐tests to determine differences at individual time points. This study was not preregistered.

## 3 RESULTS

### 3.1 Immune response

**FIGURE 2:**
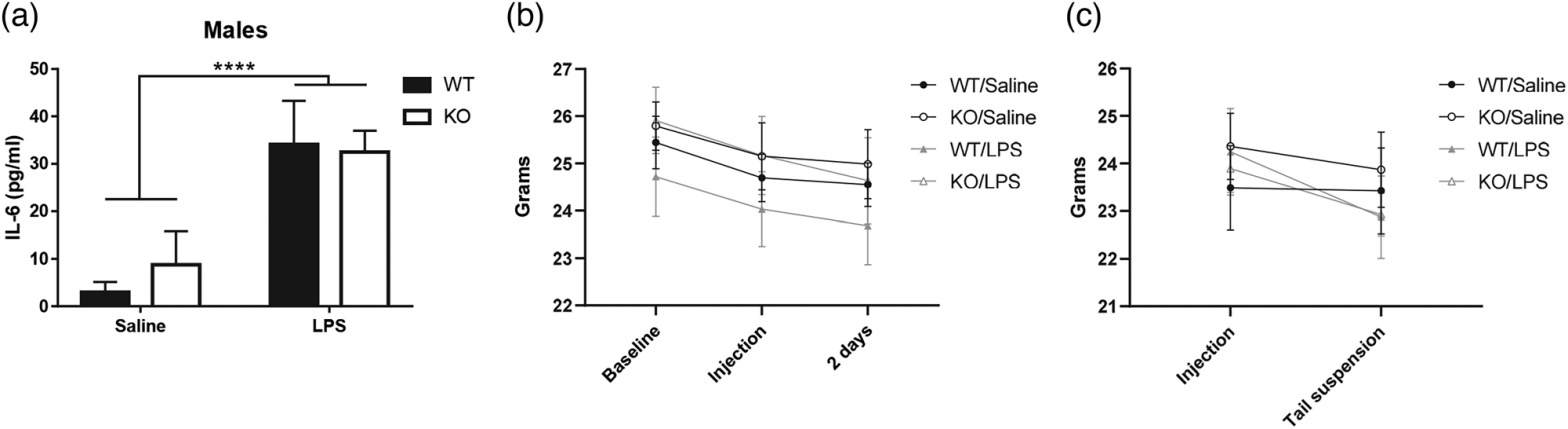
Mice physiological response to lipopolysaccharide (LPS). Plasma levels of interleukin‐6 (IL‐6) were measured 24 h after LPS administration. (a) Level of IL‐6 in males is increased in wild‐type (WT) and knockouts (KOs) mice after LPS administration (N = 7–10). (b and c) Mice weight was measured throughout the study. (b) Mice weight at baseline, injection and after wheel running test occurred. (c) Mice weight decreased from injection day to 24 h later on the tail suspension test day. Data are expressed as mean ± SEM. IL‐6 data are analyzed by two‐way analysis of variance (ANOVA). Main effect of treatment is represented by ****p < 0.0001. Weight data are analyzed by repeated‐measures two‐way ANOVA.

To confirm the immune response to LPS and assess if there were any genotype‐specific effects in the response to LPS, blood was collected 24 h after treatment and the levels of the cytokine IL‐6 were measured in the serum. A main effect of LPS treatment was identified (*F* [1,29] = 20.77, *p* < 0.0001, Figure 2a). No main effect of genotype or interaction between genotype and treatment was identified.

### 3.2 Body weight

Changes in weight of wheel running mice were examined by performing a repeated‐measures ANOVA, with a within‐subjects factor of “time” (beginning of baseline running, time of injection, 50 h after injection) and between‐subject factors of genotype and treatment. Time did not interact with treatment (*F* [2,88] = 0.59, *p* = 0.56) or genotype (*F* [2,88] = 0.06, *p* = 0.94). There was also no interaction between time, treatment, and genotype (*F* [2,88] = 0.13, *p =* 0.88). Between‐subjects effects also revealed no effect of treatment (*F* [1,44] = 0.36, *p* = 0.55), genotype (*F* [1,44] = 1.20, *p* = 0.28), or an interaction between treatment and genotype (*F* [1,44] = 0.24, *p* = 0.63) in male mice (Figure 2b).

In the cohort tested 24 h following immune stimulation, a significant interaction between time and treatment for weight was detected (*F* [1,46] = 8.69, *p* < 0.05); however, post hoc analyses revealed no discernable effects. No significant interaction between time and genotype (*F* [1,46] = 0.001, *p* = 0.98), or between time, treatment, and genotype (*F* [1,46] = 1.91, *p* = 0.17) was detected. There were also no significant differences between subject effects of treatment (*F* [1,46] = 0.17, *p* = 0.68), genotype (*F* [1,46] = 0.12, *p* = 0.73), or an interaction between treatment and genotype (*F* [1,46] = 0.30, *p* = 0.59; Figure 2c).

### 3.3 Wheel running

**FIGURE 3:**
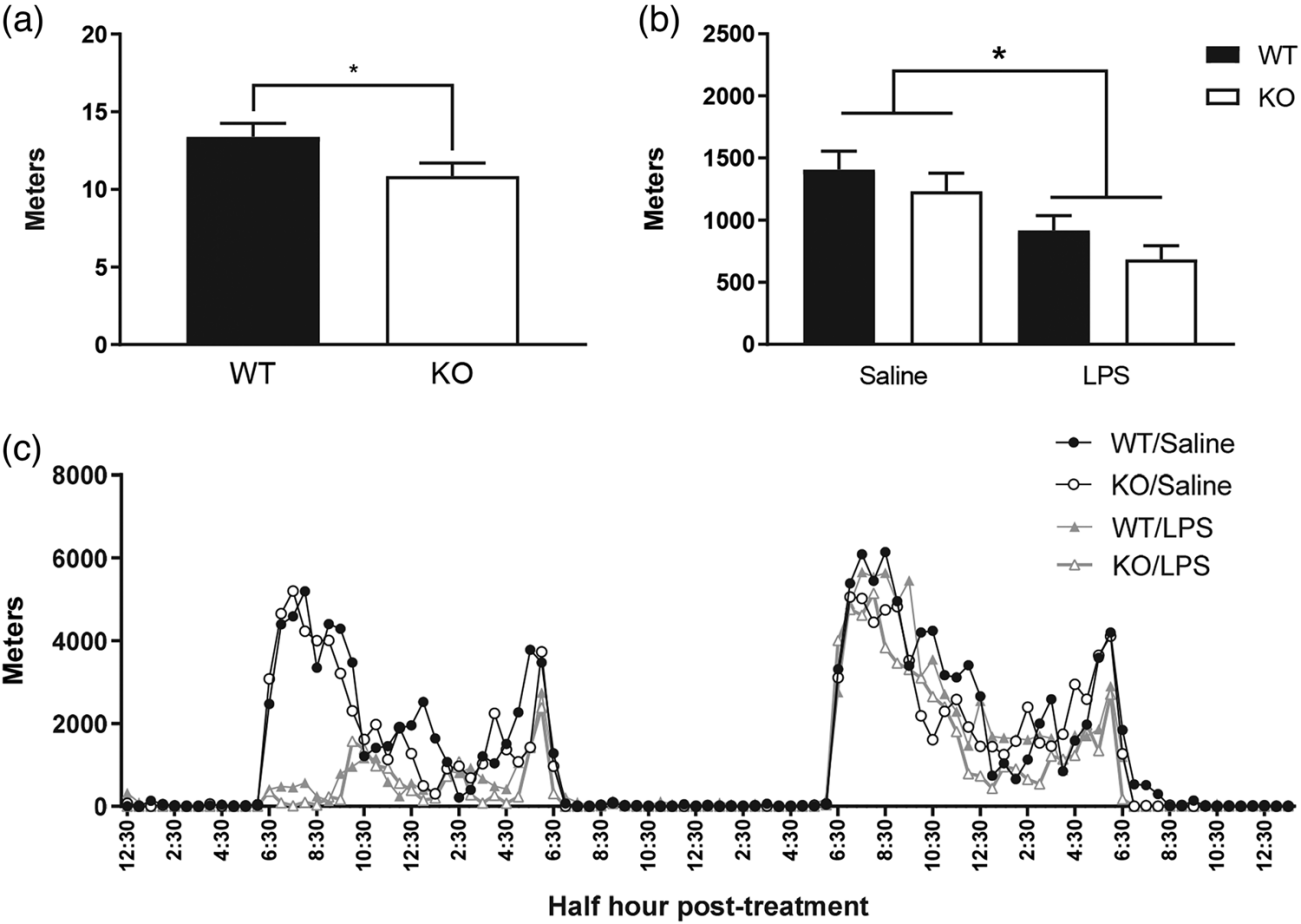
Immune stimulus with lipopolysaccharide (LPS) decreases total distance travelled in the wheel running test. (a) Baseline total distance travelled in *Fmr1* knockouts (KOs) is lower than wild‐type (WT) mice (N = 23–24). (b) LPS treatment decreased the total distance ran independent of genotype (N = 11–12). (c) Representation of wheel running across time after LPS administration. Data are expressed as mean ± SEM. Total distance ran at baseline is analyzed by *t*‐test. Total distance ran after LPS administration is analyzed by two‐way analysis of variance (ANOVA). Main effect of treatment is represented by *p < 0.05.

Prior to immune stimulation, all mice went through a period of baseline running for 4 days (96 h). Due to technical error, one WT/LPS mouse was excluded from all wheel running analyses. We examined any potential genotype differences in average distance ran (in meters) during the baseline period by performing a *t*‐test. *Fmr1* KO mice ran significantly less on average compared to WT mice (*t* [45] = 2.10, *p* < 0.05; Figure 3a).

Following administration of LPS or saline, we examined total distance ran during a testing period of ∼2 days (∼50 h). There was a significant main effect of treatment (*F* [1,43] = 15.19, *p* < 0.001), with LPS‐treated mice running significantly less compared to control mice. No significant main effect was detected for genotype (*F* [1,43] = 2.35, *p* = 0.13), nor was there a significant interaction between treatment and genotype for total distance ran (*F* [1,43] = 0.05, *p* = 0.82; Figure 3b).

We also ran a two‐way repeated‐measures ANOVA, with a within‐subjects factor of “time,” to examine potential genotype differences in voluntary running across the testing period following an immune stimulus. There was a significant interaction between time and treatment (*F* [98,4214] = 6.35, *p* < 0.001). Post‐hoc analyses revealed that mice that received LPS ran significantly less at several time points following immune stimulation, specifically at the following half‐hour increments post‐LPS or saline: 6.5, 7, 7.5, 8, 8.5, 9, 9.5, 10, 12, 12.5, 13, 16, 16.5, 17, 17.5, 36, 41.5, and 42. No significant interaction was detected for time and genotype (*F* [98,4214] = 1.20, *p* = 0.09), nor was there an interaction between time, treatment, and genotype (*F* [98,4214] = 0.86, *p* = 0.83; Figure 3c).

### 3.4 Tail suspension

**FIGURE 4:**
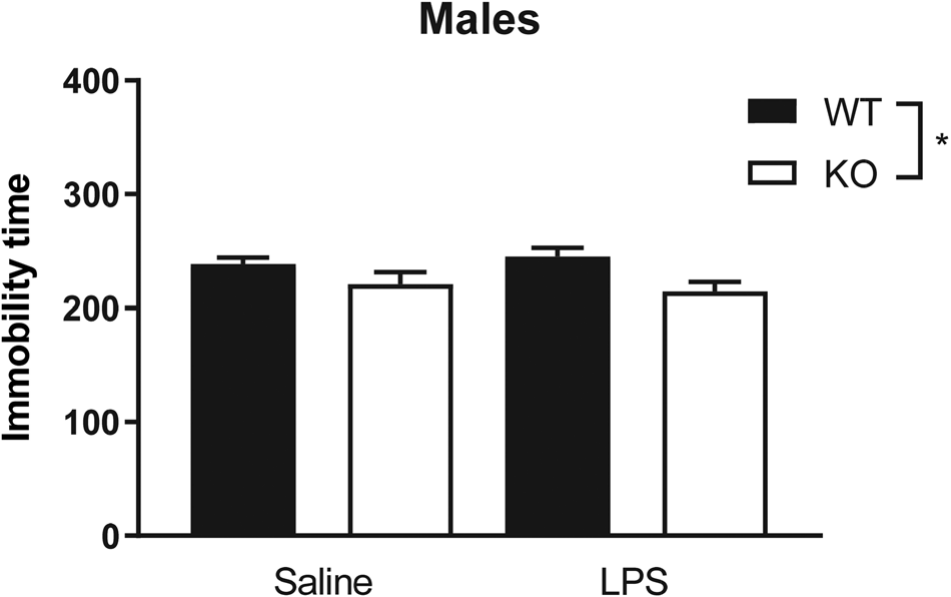
Immobility time in the tail suspension test is decreased in *Fmr1* mutant mice. Tail suspension test was performed 24 h after lipopolysaccharide (LPS) stimulation. Total immobility time was decreased in *Fmr1* KO independent of LPS treatment (N = 11–14). Data are expressed as mean ± SEM and analyzed by two‐way analysis of variance (ANOVA). Main effect of genotype is represented by *p < 0.05.

Twenty‐four hours after LPS administration, tail suspension test was performed. There was no effect of treatment (*F* [1,46] = 0.00, *p* = 0.98). However, there was a significant effect of genotype (*F* [1,46] = 8.19, *p* < 0.05), with *Fmr1* KO mice spending significantly less time immobile during the task compared to WT mice. No interaction between treatment and genotype was detected (*F* [1,46] = 0.61, *p* = 0.44; Figure 4).

## 4 DISCUSSION

We and others have previously shown that *Fmr1* mutant mice exhibit an altered physiological immune response (Hodges et al., 2020; Parrott et al., 2021). In the present study, we investigated whether male *Fmr1* KO mice also present with an altered behavioral response to inflammation using the bacterial endotoxin LPS as an immune stimulus. To assess alterations in sickness and depressive‐like behavior in response to LPS, mice were tested in the wheel running and tail suspension test.

LPS is a widely used inflammatory stimulus that can induce sickness behavior and depressive‐like behavior that varies in severity according to the dose administered (Lasselin et al., 2020; Xu et al., 2007). We measured the levels of the proinflammatory cytokine IL‐6 in the serum 24 h after LPS treatment to confirm that the dose of LPS (0.1 mg/kg) used in our study induced an immune response in both genotypes. LPS caused an increase in IL‐6 in all treated groups as expected. Another consequence of systemic inflammatory stimuli with LPS is weight loss. Weight was measured to assess if weight loss could account for the behavioral deficits we identified, as it is possible that a reduction in weight resulted in fatigue which could impact wheel running. Treated mice did not present a significant weight loss in response to LPS when measured at 1 and 2 days after the immune stimulus occurred.

Sickness behavior was identified in the wheel running test by decreased activity in comparison to controls. KO mice ran less than WT mice during baseline recordings. After administration of LPS, however, both genotypes had a similar reduction in total distance ran when compared to non‐treated controls. There was no genotype effect indicating that *Fmr1* did not play a role in the effect LPS had on the wheel running test. The fact that *Fmr1*‐deficient mice presented with a similar sickness response to controls following LPS administration suggests that increased levels of IL‐6 did not alter their behavioral sickness response in the wheel running test. Our previous study that found differences in the immune response between *Fmr*‐deficient mice and controls used higher dose of LPS (0.33 mg/kg) than we used in the current study (0.1 mg/kg) and used a different background strain (Fvb/NJ). Hodges et al. did not find alterations in sickness response measured in the food burrowing test that was suggestive of a potential flooring effect since both genotypes presented a low burying behavior in response to LPS. Here, using a different dose of LPS and different background strain (C57BL/6), we also showed no alterations in sickness behavior in *Fmr1* KO mice. Together, our studies strongly suggest that although the KO mice can present an altered immune response, these alterations may not influence sickness behaviors. A study directly comparing the immune response and how immune insults influence behavior in different *Fmr1* KO background strains would clarify the similarities and differences found in our studies.

Acute administration of LPS can induce depressive‐like behavior in rodents that can be measured by immobility time in the tail suspension test (Biesmans et al., 2013; Lasselin et al., 2020). We found that mice deficient in the *Fmr1* gene display decreased immobility time when compared to controls. These findings indicate that the *Fmr1* mutant mice present with altered behavioral despair/helplessness in the tail suspension test. These mice exhibit increased activity when compared to WT mice which is consistent with other studies that have found *Fmr1* mutant mice to be hyperactive in a variety of behavioral paradigms (Nolan et al., 2017; Saré et al., 2016). In the present study, we did not find any effect of LPS on immobility time. Previous studies that have used higher doses of LPS, such as 0.33 mg/kg and 0.83 mg/kg, have consistently found increased immobility in response to the immune stimulus (Martin et al., 2014; O'Connor et al., 2009). This suggests that the dose used here may have been too low to induce an increase in immobility time in response to LPS during the tail suspension test. Future experiments should examine depressive‐like behavior in additional paradigms that are not as dependent on baseline activity levels. Mice could be tested in a forced swim task or in a sucrose preference test which examines anhedonia, one of the core symptoms of depression.

Together, these data suggest that *Fmr1* mutant mice do not present with altered sickness behavior in response to a lower dose of LPS, such as that used in our study (0.1 mg/kg). We used a low dose of LPS in our study because we wanted to assess not only a potential altered sickness response, but if *Fmr1* KOs could have a lower threshold to the effects of LPS in the behavior test used in the present study. However, we did not see any interactions between the LPS treatment and genotypes. Previous studies that have identified an altered immune response in *Fmr1* mutant mice used LPS doses higher than 0.1 mg/kg. Hence, it is possible that a higher dose is necessary to reveal differences in the behavioral sickness response between controls and *Fmr1* mutant mice. Another important finding in our study is that although the mice did have increased peripheral levels of IL‐6, they did not present alterations in behavioral despair/helplessness in the tail suspension test 24 h after LPS treatment. Further studies are necessary to better understand this response in the tail suspension test to determine the threshold at which differences may exist in the sickness response of *Fmr1‐*deficient mice.

## AUTHOR CONTRIBUTIONS

Formal analysis, validation, writing‐original draft, supervision, and writing‐review and editing: Danielle Santana‐Coelho. Conceptualization, methodology, data curation, formal analysis, validation, supervision, and writing‐original draft: Samantha L. Hodges. Investigation, visualization, and writing‐review and editing: Saul I. Quintero. Investigation, visualization, and writing‐review and editing: Paige D. Womble. Formal analysis, supervision, visualization, and writing‐review and editing: D. Greg Sullens. Investigation, visualization, and writing‐review and editing: David A. Narvaiz. Investigation, visualization, and writing‐review and editing: Rebecca Herrera. Resources and methodology: Melanie J. Sekeres. Conceptualization, formal analysis, funding acquisition, methodology, project administration, supervision, writing‐original draft, and writing‐review and editing: Joaquin N. Lugo.

## ACKOWLEDGMENTS

The research was funded by NIH NINDS grant R15NS088776 (Joaquin N. Lugo). The graphical abstract was created using Biorender.

## CONFLICT OF INTEREST STATEMENT

The authors declare no conflict of interest.

### PEER REVIEW

The peer review history for this article is available at https://publons.com/publon/10.1002/brb3.3142.

## Data Availability

The data that support the findings of this study are available from the corresponding author upon reasonable request.
